# Enhanced efficiency of carbon based all perovskite tandem solar cells via cubic plasmonic metallic nanoparticles with dielectric nano shells

**DOI:** 10.1038/s41598-024-78165-0

**Published:** 2024-11-02

**Authors:** Amir Hossein Mohammadian Fard, Samiye Matloub

**Affiliations:** Quantum Photonics Research Lab (QPRL), Faculty of Electrical and Computer Engineering, University of Tabriz, Tabriz, 5166614761 Iran

**Keywords:** All-perovskite tandem solar cells, High efficiency, Solar energy and photovoltaic technology, Nanoparticles

## Abstract

This study investigates a carbon-based all-perovskite tandem solar cell (AP-TSC) with the structure ITO, SnO₂, Cs₀.₂FA₀.₈Pb(I₀.₇Br₀.₃)₃, WS₂, MoO₃, ITO, C₆₀, MAPb₀.₅Sn₀.₅I₃, PEDOT: PSS, Carbon. The bandgap configuration of the cell is 1.75 eV/1.17 eV, which is theoretically limited to 36% efficiency. The effectiveness of embedding cubic plasmonic metallic nanoparticles (NPs) made of Gold (Au) and Silver (Ag) within the absorber layers to eliminate the requirement for thicker absorber layers, decrease manufacturing costs and Pb toxicity is demonstrated in our analysis. This analysis was conducted using 3D Finite Element Method (FEM) simulations for both optical and electrical calculations. Prior to delving into the primary investigation of the tandem structure, a validation simulation was conducted to demonstrate the accuracy and reliability of the simulations. Notably, the efficiency mismatch observed during the validation simulation, specifically in relation to the incorporation of metallic nanoparticles (NPs), amounted to a mere 0.01%. To mitigate the potential issues of direct contact between metallic NPs and perovskite materials, such as increased thermal and chemical instability and recombination at the NP surface, a 5 nm dielectric shell was applied to the NPs. The incorporation of cubic core-shell Ag NPs resulted in a 15.32% enhancement in short-circuit current density, from 16.39 mA/cm² to 18.90 mA/cm², and a 15.68% increase in overall efficiency, from 26.9 to 31.12%. This research paves the way for the integration of core-shell metallic NPs in AP-TSCs, highlighting a significant potential for efficiency and stability improvements. In a dedicated section the band alignment of the sub-cell was addressed. Additionally, a thermal investigation of the proposed tandem structure was conducted, demonstrating the robustness of the proposed AP-TSC. Finally, the sensitivity analyses related to input parameters and the challenges associated with large-scale fabrication of the proposed AP-TSC were extensively discussed.

## Introduction

The detailed balance limit for single-junction solar cells (SJ-SCs) is a critical challenge that restricts their efficiency. This limit, known as the Shockley-Queisser limit, caps the efficiency of SJ-SCs at approximately 33%^1,2^. SJ-SCs are unable to absorbing photons with energies below the bandgap of the absorber layer, and photons with energies exceeding the bandgap result in phonon interactions and thermal losses^3^. Tandem solar cells (TSCs) offer a promising solution to this problem by stacking multiple absorber materials. The theoretical efficiency limits for TSCs are 50%, 54%, and 65% for three, four, and an infinite number of sub-cells, respectively^4^. In a TSC, the top sub-cell is designed with a wide-bandgap (WBG) absorber to absorb photons with higher energies and shorter wavelengths, while the bottom sub-cell incorporates a narrow bandgap (NBG) absorber to absorb photons at longer wavelengths. This configuration allows TSCs to absorbs broader spectrum of sunlight, resulting in higher efficiencies and reduced thermalization losses due to effective absorption of the near-infrared region by NBG materials^5^.

The first experimental investigation of TSCs dates back to 1978, when a tandem structure utilizing AlGaAs / GaAs as absorber materials was studied, achieving an of efficiency of 9%^6^. Since then, significant advancements have been made, with efficiencies reaching 32.6% for GaInAsP / GaInAs, 32.8% for GaInP / GaAs, and 37.9% for a three-junction GaInP/GaAs/GaInAs tandem structure^7–9^. It is evident that III-V solar cells exhibit the highest efficiencies among all photovoltaic technologies, but their high fabrication costs have limited their market share. In the 1980s, silicon emerged as an alternative for substrates with narrower bandgap in tandem structure to reduce manufacturing costs. Nonetheless, significant differences in thermal expansion coefficients and lattice constants between Si and III-V materials posed challenges^10^. Consequently, researchers sought better alternatives to achieve high efficiency and lower fabrication costs in tandem structures. Organic and inorganic perovskite materials have emerged as the most promising candidates to replace traditional absorber materials such as silicon and III-V semiconductors. These materials have attracted considerable attention due to their high efficiency, tunable optical band gap, high absorption, low exciton binding energy, long carrier lifetime, and low-cost solution processability compared to previous generations of solar cells^11–15^. Commonly used materials in perovskite-based structures include MAPbI₃^16^, MAPbBr₃^17^, MASnI₃^18^, Cs₂AgBiBr₆^19^, and CsPbI₃^20^, all of which have extensive attention in the research community. All-perovskite TSCs (AP-TSCs) typically consist of a NBG (*Eg* ≈1.2–1.3 eV) mixed tin-lead (Sn-Pb) bottom sub-cell to absorb longer wavelengths and a WBG, (*Eg*​≈1.7–1.9 eV) top sub-cell to absorb shorter wavelengths^21–23^.

In most perovskite-based TSCs (P-TSCs), metals such as silver (Ag) and gold (Au) are commonly employed as the back contact electrode. However, this approach has several disadvantages, including the necessity for thermal evaporation coating, which significantly increases the manufacturing cost of tandem structures. Consequently, the commercialization of P-TSCs is hampered^24^. To address this issue, the current research explores the use of carbon as the back contact electrode. Nonetheless, utilizing carbon in an AP-TSC structure can lead to reduced efficiency. One method to enhance the absorption of the tandem structure is the implementation of thicker absorber layers; however, this technique poses experimental challenges and can result in increased losses due to the recombination process^25,26^.

An alternative to utilizing thicker absorber layers for maximizing absorption in tandem structures is the light trapping technique. Light trapping involves increasing the optical thickness of device to be several times greater than its physical thickness, thereby enhancing the interaction of the solar cell with light. This results in increased absorption in the perovskite layer due to local field enhancement. The increase in absorption leads to a higher generation of electron-hole pairs, consequently, improving both efficiency and photocurrent^27^. One effective method for implementing light trapping is through the incorporation of plasmonic nanoparticles (NPs) at various locations, using different types of metals and geometry shapes^28^. Among these NPs, spherical NPs have been extensively studied in PSCs and are often embedded within the perovskite layer itself^29^. The interaction of incident light with metallic NPs induces surface oscillation of free electrons at the plasma resonance frequency, resulting in localized surface plasmon resonance (LSPR). Understanding the impact of incident light on metallic NPs and the interaction between incident light and the free electrons in metals is crucial for harnessing the benefits of LSPR in enhancing solar cell performance.

It is noteworthy that, based on effective medium theory and the Yablonovitch limit, the embedding of metallic NPs increases the effective refractive index of absorber material. This enhancement depends on the type and morphology (shape and thickness) of metallic NPs embedded in the absorber layer, significantly boosting the absorption in the perovskite absorber layer^30^. In recent years, several studies have focused on embedding plasmonic metallic NPs in the SJ-PSC. For instance, the incorporation of two spherical gold NPs in the absorber layer resulted in a 14% increase in efficiency^31^. Another study embedded three geometric types of Platinum NPs within the perovskite layer, observing a 22% enhancement in efficiency^32^. Quadrilateral cluster NPs composed of various metals achieved a substantial 28.72% enhancement in photocurrent, reaching 22.28 mAcm^− 2^^33^. More recently, research has investigated the influence of embedding plasmonic NPs made from various materials in the NBG absorber layer of AP-TSC, achieving a short-circuit current of 16.37 mAcm^− 2^ with efficiency of 33.37%^34^.

One of the significant challenges in the manufacture of perovskite-based solar structures, such as SJ-PSCs and P-TSCs, is the decomposition of perovskite materials. When photons interact with free electrons in metallic NPs, some electrons do not contribute to increased absorption in the absorber materials and remain on the NP surface. This phenomenon causes heating at the surface of metallic plasmonic NPs during phonon radiation and increases recombination in the presence of metallic NPs^35^. Additionally, the decomposition of NPs decreases efficiency^33^. To overcome this issue, adding a high stability dielectric material to metallic NPs is an effective solution. Silicon dioxide (SiO₂) is the most commonly used dielectric material, as its use prevents direct contact between the perovskite absorber material and metallic NPs, enhancing NPs stability. Furthermore, embedding core-shell NPs also reduces the toxicity of lead-based absorber layers^36^.

The short-circuit current of TSC is approximately equal to that of the WBG sub-cell, while the open-circuit voltage corresponds to the sum of open-circuit voltage of sub cells. This underscores the critical importance of the short-circuit current in the WBG sub-cell for the overall performance of TSC. The incorporation of plasmonic metallic NPs presents a promising solution to enhance the efficiency of AP-TSCs toward their efficiency limits. Given the limitation of laboratory resources and the time-consuming nature of the manufacturing process, simulations are essential for developing effective AP-TSCs. While the three-dimensional finite difference time domain (3D-FDTD) method is recognized for its accuracy in optical calculations, less precise 1D simulations have often been used for electrical properties. To improve precision and achieve more reliable results, the 3D-finite element method (3D-FEM) method was employed in this study. This method was applied to both optical and electrical simulations, ensuring that the complexities inherent in AP-TSCs were more accurately represented.

While the incorporation of metallic NPs has been extensively studied in SJ-PSCs, research focusing on their integration within AP-TSCs remains limited. Most studies conducted to date have employed spherical NPs to enhance light absorption in SJ configurations^37^. Nevertheless, the potential for plasmonic enhancements in tandem structures remains underexplored, with only a few reports addressing spherical NPs in AP-TSCs^38,39^. In contrast to these previous studies, the current work introduces cubic core-shell NPs, which have been demonstrated to significantly enhance device performance. The sharp edges of cubic NPs offer improved local field effects, leading to increased absorption and electron-hole pair generation^40,41^. To our knowledge, this represents the first investigation into the use of cubic core-shell NPs in AP-TSCs, resulting in a notable 15.32% increase in short-circuit current density and a 15.68% improvement in overall efficiency.

In this research, an AP-TSC is investigated using the 3D-FEM to develop a device that is low in toxicity, cost-effective, eco-friendly, easy to fabricate, features thinner absorber layers, and exhibits high efficiency. The AP-TSC structure incorporates absorber layers with bandgaps of 1.75 eV and 1.17 eV, chosen for their high efficiency limits, which can reach up to 36%. Initially, each sub-cell is examined individually. Subsequently, cubic metallic plasmonic core-shell NPs composed of Au and Ag with a SiO_2_ shell are embedded in the absorber layers, to assess the impact of these metallic NPs on the optical and electrical characteristics of the sub-cells, including absorption, electron-hole generation, short-circuit current, and overall efficiency. After individual analysis, the WBG sub-cell is stacked on NBG sub-cell to form the tandem structure, incorporating a specific recombination layer. Metallic core-shell NPs are embedded in both sub-cells, and their thickness is varied to determine the optimal thickness that maximizes efficiency. Current matching is achieved by first adjusting the thickness of metallic NPs and then modifying the thickness of the absorber layers.

This study is organized into five main sections. The second section details the overall structure of the AP-TSC with metallic core-shell NPs. The third section thoroughly investigates the optical and electrical formulation for accurate analysis of the AP-TSC. The fourth section presents the results of optical and electrical simulations, including diagrams of optical properties such as absorption and generation, as well as electrical parameters like the J-V curve and band diagram. The impact of temperature on the performance of the AP-TSC is discussed in this section. Moreover, sensitivity analysis of input parameters and the challenges in scaling from simulation to real-world applications are investigated. Finally, the fifth section summarizes and concludes the research, highlighting key findings.

## **The structure of the proposed AP-TSC**

In this research, the proposed AP-TSC comprises several distinct layers, each contributing specifically to the optical and electrical behavior of the device. The initial layer is transparent conductive oxide (TCO) layer, employed to minimize reflection losses, enhance absorption and thereby improve the overall efficiency^42^. Indium tin oxide (ITO) is selected for TCO anti-reflection (AR) layer. The subsequent layer is the Electron Transport Layer (ETL) for the top sub-cell, which prevents hole migration and facilitates the transfer of electrons to the external electrode. Photovoltaic performance is significantly influenced by charge mobility, energy level alignment, defect states, morphology, and interfacial properties^43^. Among the various ETL candidates, SnO_2_ is preferred due to its minimal open-circuit voltage loss within the solar structure. Femtosecond transient absorption measurements have demonstrated SnO_2_’s high electron extraction efficiency^44^. With a large band gap of approximately 3.6 eV, SnO_2_ permits the transmission of most visible light while blocking UV absorption, thus protecting the cell from UV exposure^45,46^. Additionally, SnO₂’s bulk electron mobility is two orders of magnitude higher than that of TiO₂^47^, and it can be processed at low temperatures, making it suitable for large-scale commercialization^48,49^.

The next layer is the WBG perovskite absorber layer, composed of Cs_0.2_FA_0.8_Pb(I_0.7_Br_0.3_)_3_ with a band gap of 1.75 eV, designed to absorb high-energy photons. Following this is a very thin WS_2_ transition metal dichalcogenide (TMD) layer, which enhances the absorption of WBG absorber layer in shorter wavelength range, reduces the recombination at perovskite interface, and increase the short-circuit current of WBG sub-cell. The subsequent layer is the hole transport layer (HTL) for the top sub-cell, comprised of MoO_3_, selected for its exceptional optical and electrical properties, stability, and favorable interface characteristics^50^. Next, the interconnection layer (ICL) is introduced to provide a series connection between the two sub-cells. This layer must ensure minimal absorption and high photon transmission to reduce absorption losses in NBG sub-cell. ITO is employed in this layer with minimal thickness. This meticulously designed layer structure aims to optimize the efficiency and performance of the AP-TSC by enhancing each layer’s role in the device’s overall function.

In the bottom sub-cell, the sixth layer serves as the ETL for NBG sub-cell. C_60_, recognized for its high electron mobility and suitability for low-temperature evaporation process, is widely utilized as an ETL in PSCs. At the interface between the perovskite and the C_60_ layer, C_60_ facilitates efficient charge transport while reducing trap states density. Furthermore, C_60_ contributes to the long-term performance and stability of PSCs. The incorporation of C_60_ in the ETL provides an opportunity for developing high-performance and flexible photovoltaic devices with a regular n-i-p architecture. By leveraging C_60_ in the AP-TSC, improved efficiency and flexibility are achievable, offering promising prospects for advancing solar energy technologies^51–54^. Subsequently, a NBG solar cell with a band gap of 1.17 eV (MAPb_0.5_Sn_0.5_I_3_) is integrated to absorb longer wavelength photons and extend the absorption range of the tandem structure.

The selection of bandgaps for the perovskite layers, specifically 1.75 eV and 1.17 eV, is justified by the efficiency limit based on the Detailed Balance Limit, which stands at 36% for these values^55^. This theoretical upper limit for efficiency underscores the strategic importance of these bandgaps, providing a robust foundation for potential enhancements through various optimization methods. Aligning the bandgaps of the perovskite layers with this efficiency limit enables the exploration of different strategies and techniques aimed at further improving the overall efficiency of the tandem structure. This strategic selection not only maximizes the performance of the AP-TSC but also underscores the significance of incorporating theoretical efficiency limits into device design. Plasmonic metallic NPs composed of Ag and Au, each with a dielectric SiO_2_ shell layer, are incorporated into the absorber layers. The inclusion of these NPs aims to enhance light absorption through LSPR, thereby boosting the overall efficiency of the AP-TSC.

**Fig. 1:**
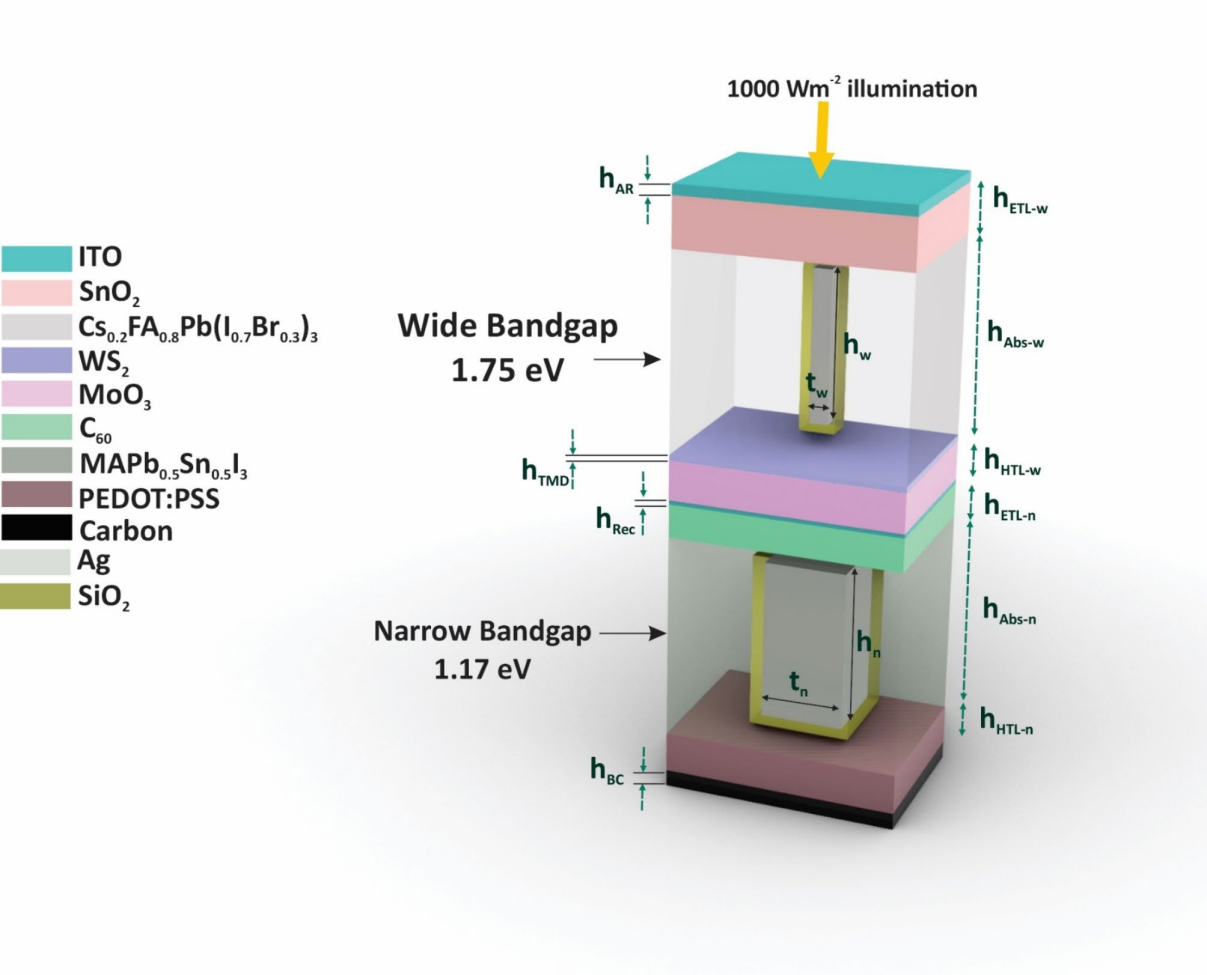
Schematic Representation of the Proposed AP-TSC Structure. This image was created using Rhino, version 8, available at [https://www.rhino3d.com].

The subsequent layer is the HTL, composed of PEDOT: PSS. This material is chosen for its high and adjustable conductivity, low-temperature solution processability, mechanically flexibility, and thermal stability, making it suitable for using as an HTL in the AP-TSC structure^56,57^. This growing interest in PEDOT: PSS has been matched by significant investments, leading to the expanded market presence. Finally, to reduce fabrication costs and enhance stability, a carbon back contact is used instead of metallic contacts^58^. Although this choice may result in a slight reduction in efficiency, the improved stability compensates for it, and additional enhancements can be pursued through alternative methods. The incorporation of core-shell metallic NPs is proposed as a means to address the efficiency reduction associated with the use of a carbon back electrode. The overall structure of the proposed AP-TSC is illustrated in Fig. 1, with the thickness of each sublayer detailed in Table 1. This comprehensive framework aims to optimize the efficiency and performance of AP-TSCs.

**Table 1:** The parameters of simulation.

Parameter	Description	Material	Thickness (nm)
h_AR_	Thickness of antireflection layer	ITO	65
h_ETL−w_	Thickness of electron transfer layer for top sub-cell	$\:{\text{S}\text{n}\text{O}}_{2}$	100
h_Abs−w_	Thickness of wide bandgap absorber layer	Cs_0.2_FA_0.8_Pb(I_0.7_Br_0.3_)_3_	1200(NPs),760(without NPs)
h_HTL−w_	Thickness of hole transfer layer for top sub-cell	$\:{\text{M}\text{o}\text{O}}_{3}$	150
h_Rec_	Thickness of recombination layer	ITO	17
w_h_	Period of structure	-	200
h_ETL−n_	Thickness of electron transfer layer for bottom sub-cell	C_60_	100
h_Abs−n_	Thickness of narrow bandgap absorber layer	$\:{\text{M}\text{A}\text{P}\text{b}}_{0.5}{\text{S}\text{n}}_{0.5}{\text{I}}_{3}$	600,450(Ag),430(Au)
h_HTL−n_	Thickness of hole transfer layer for bottom sub-cell	PEDOT: PSS	50
h_BC_	Thickness of back contact layer	Carbon	80
t_n_	Height and width of NBG metallic NPs	-	170
t_w_	Height and width of WBG metallic NPs	-	70(Ag),60(Au)
h_w_	The thickness of WBG metallic NPs	Ag, Au	190(Au),500(Ag)
h_n_	The thickness of NBG metallic NPs	Ag, Au	300
z_np_	The distance between core shell NPs and HTL	-	50
t_Shell_	The thickness of shell layer	SiO2	5
h_TMD_	The thickness of TMD layer	WS_2_	12

## Modeling and simulation approaches for AP-TSCs

**Fig. 2:**
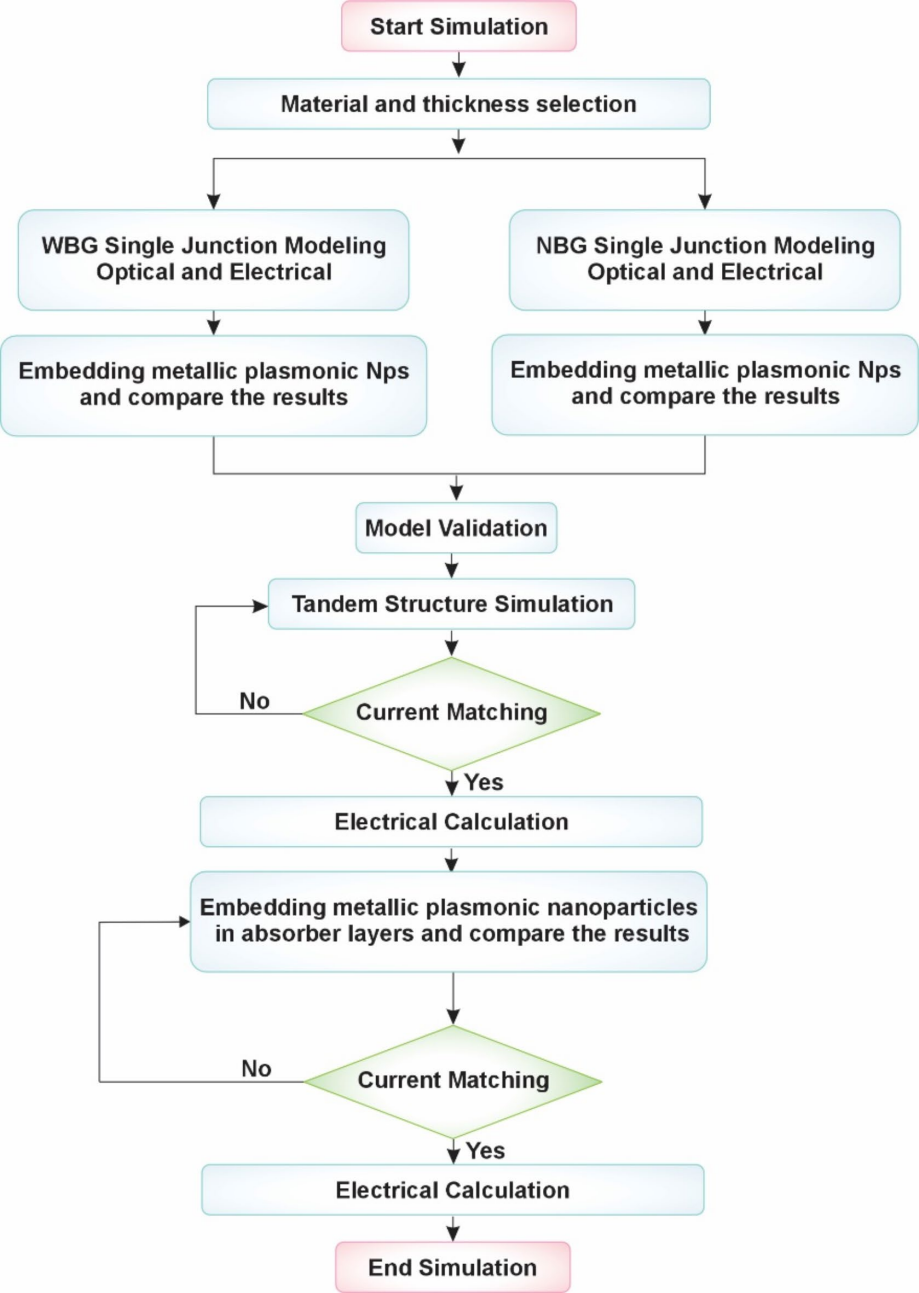
Flowchart for investigation of proposed all-perovskite tandem structure.

The overall methodology required to investigate the proposed AP-TSC is summarized in Fig. 2. Initially, all optical and electrical parameters required for the study were collected from relevant literature sources^50,55,59–65^. Each WBG and NBG sub-cell was then examined individually as SJ-PSC, with a focus on their optical and electrical characteristics. Subsequently, metallic plasmonic NPs with dielectric nano-shells were incorporated into the absorber layers of each sub-cell to evaluate the impact of plasmonic enhancement on their performance.

Following the individual investigation, the WBG sub-cell was stacked on top of the NBG sub-cell with specified ICL, thus forming the tandem structure. This tandem structure was simulated, and optical and electrical characteristics were computed under precise boundary conditions. In a manner similar to the individual investigation of sub-cells, plasmonic metallic NPs composed of Ag and Au with dielectric nano-shells were incorporated into the tandem structure. The effect of these metallic NPs was thoroughly investigated. Subsequently, the thickness of NPs was varied within specified range to determine its influence on cell performance. Finally, current matching was achieved by modifying both the NPs thickness and the absorber layer thickness, ensuring optimal performance by balancing the photocurrents generated in the tandem structure.

It is noteworthy that both optical and electrical analyses were performed using 3D-FEM approach with precise boundary conditions. During the optical simulation phase, key properties, including the electron-hole generation rate, absorption spectrum, transmission and reflection characteristics, and electric field distribution, were calculated for the tandem structure. These characteristics were essential in determining the electrical properties under defined conditions, providing a thorough understanding of the tandem structure’s overall performance.

Validation is a critical step in ensuring the reliability and accuracy of simulation results. Without proper validation, the insights gained from modeling might not accurately reflect real-world behaviors and phenomena. By comparing the simulated results with experimental data, the robustness and credibility of the findings can be confirmed. In this study, validation is essential not only for verifying the optical and electrical characteristics of the AP-TSC but also for assessing the impact of various enhancements, such as plasmonic NPs integration. Thus, the validation process strengthens the confidence in the proposed design and simulation methods, ensuring that they provide practical and actionable insights for future advancements in TSC.

### A. Modeling and simulation of AP-TSCs

To comprehensively understand the optical characteristics of the proposed AP-TSC, a detailed investigation of a unit cell was conducted. The unit cell, defined by specified width and length (w_h_), was thoroughly examined. A scattering boundary condition was applied on the top surface of the ITO AR layer to simulate the AM1.5 solar spectrum within the wavelength range of 300–2000 nm. The tandem structure designed such that the WBG sub-cell absorbing high-energy photons with shorter wavelengths, while the NBG sub-cell absorbs photons with longer wavelengths. This configuration enables the absorption of a broader range of wavelengths, contributing to the high efficiency of the tandem structure.

The optical behavior of sub-cells— including absorption, electron-hole pair generation, transmission, reflection, and electric-field distribution was thoroughly studied. Perfectly matched layers (PMLs) were applied along the sun irradiation direction of solar irradiation, while a periodic boundary condition was set in the orthogonal direction to replicate the unit cell ensuring the reliable investigation of optical behavior. Initial simulations were performed without metallic NPs, followed by repeated simulations with the incorporation of metallic NPs, allowing for a comparative analysis of their impact on performance. Notably, these investigations were carried out separately for each WBG and NBG sub-cell before being applied to the tandem structure, resulting in separate results and spectra for each sub-cell.

Maxwell’s equations were initially solved using 3D-FEM method to examine the optical characteristics of the proposed AP-TSC. This approach determined the intensity of electric field$\:\:\left|E(r,\lambda\:)\right|$ at any specified location within proposed tandem structure, allowing for the calculation of key optical characteristics, including absorption, electron-hole generation, transmission, and reflection were calculated. Equation (1) was then employed to compute the absorption spectrum in each of the WBG and NBG absorber layers.

1 $$A(r,\omega )=\frac{1}{2}\frac{{\omega {\varepsilon _0}\operatorname{Im} (\varepsilon (r,\omega )){{\left| {E(r,\lambda )} \right|}^2}}}{{{P_{in}}}}$$

In this equation, *r* represents a specific location in the all-perovskite tandem structure, while *ω* denotes the angular frequency. $\:{\epsilon\:}_{0}$ and *ε* correspond to the dielectric constants of the vacuum and the environment, respectively, and these values will vary for each absorber layer based on its own refractive index ($\:\epsilon\:\left(r,\omega\:\right)=Re\left(\epsilon\:\left(r,\omega\:\right)\right)+iIm\left(\epsilon\:\left(r,\omega\:\right)\right)$). P_in_ represents the power of incident light under solar irradiation, which based on AM1.5G equals to 1000 Wm^− 2^. Equations (2) and (3) define the total absorption for WBG and NBG absorber layers, respectively, which is calculated by integrating the absorption *A*(*r*, *λ*) over the volume of the absorber layers.

2 $${A_{wtotal}}=\int_{{{h_{BC}}+{h_{HTL - n}}+{h_{Abs - n}}+{h_{ETL - n}}+{h_{\operatorname{Re} c}}+{h_{HTL - w}}+{h_{TMD}}}}^{{{h_{BC}}+{h_{HTL - n}}+{h_{Abs - n}}+{h_{ETL - n}}+{h_{\operatorname{Re} c}}+{h_{HTL - w}}+{h_{TMD}}+{h_{Abs - w}}}} {\int_{{ - \frac{{{w_h}}}{2}}}^{{\frac{{{w_h}}}{2}}} {\int_{{ - \frac{{{w_h}}}{2}}}^{{\frac{{{w_h}}}{2}}} {A(x,y,z,\lambda )dxdydz} } }$$

3 $${A_{ntotal}}=\int_{{{h_{BC}}+{h_{HTL - n}}}}^{{{h_{BC}}+{h_{HTL - n}}+{h_{Abs - n}}}} {\int_{{ - \frac{{{w_h}}}{2}}}^{{\frac{{{w_h}}}{2}}} {\int_{{ - \frac{{{w_h}}}{2}}}^{{\frac{{{w_h}}}{2}}} {A(x,y,z,\lambda )dxdydz} } }$$

These integrals compute the total absorption throughout the volume of each of the absorber layers individually. Using similar integrals, the absorption of each layer can be calculated. The filtered sunlight irradiation spectrum that the bottom sub-cell is exposed to can be calculated using Eq. (4).

4 $${S_{filtered}}(\lambda )=S(\lambda )[\sum\limits_{{k=1}}^{5} { - ({a_k}(\lambda ) - {t_k})} ]$$

In Eq. (4) $\:{S}_{filtered}\left(\lambda\:\right)$ is the filtered spectrum of sunlight, $\:S\left(\lambda\:\right)$ is the AM1.5G sunlight irradiation spectrum, $\:{a}_{k}\left(\lambda\:\right)$ is absorption of each layer, k represents 1, 2, 3, 4 and 5 for the different layers of top-cell and t is the thickness of specified layer. In this article, the structure is continuously simulated, and the Eq. (4) has not been used for optical calculations. From Eq. (2) and Eq. (3), the electron-hole generation pair rate can be calculated individually using Eqs. (5) and (6),

5 $${G_{wbg}}(x,y,z,\omega )=\frac{1}{{\hbar \omega }}{A_{wbg}}(x,y,z,\omega )$$

6 $${G_{nbg}}(x,y,z,\omega )=\frac{1}{{\hbar \omega }}{A_{nbg}}(x,y,z,\omega )$$

where $\:{G}_{wbg}\left(x,y,z,\omega\:\right)$ introduces the generation rate for WBG absorber layer, and $\:{G}_{nbg}\left(x,y,z,\omega\:\right)$ represents the generation rate of electron-hole pairs for NBG absorber layer. Here, ℏ is the reduced Planck constant. These equations enable the calculation of the generation rate at any specific location within the structure, which is crucial for determining the electrical behavior of the proposed structure, such as the short-circuit current. Additionally, the electron-hole generation pairs are directly influenced by the fundamental parameters of the absorber layer and the squared electric field $\:{\left|E(r,\lambda\:)\right|}^{2}$. To achieve the total electron-hole generation rate for each absorber layers, Eq. (7) is performed for WBG, and Eq. (8) is utilized for NBG absorber layer.

7 $${G_{wtot}} = \int_{300{\text{~}}nm}^{1100{\text{~}}nm} {{G_{wbg}}(x,y,z,\lambda )} d\lambda$$

8 $${G_{ntot}} = \int_{300{\text{~}}nm}^{1100{\text{~}}nm} {{G_{nbg}}(x,y,z,\lambda )} d\lambda$$

Equation (7) and Eq. (8) were utilized to determine the electron-hole pair generation rates necessary for the semiconductor physics calculations. It is important to note that throughout the optical calculations, only the absorption of the Cs₀.₂FA₀.₈Pb(I₀.₇Br₀.₃)₃ and MAPb₀.₅Sn₀.₅I₃ materials in the absorber layers was considered for calculating the generation rate of electron-hole pairs. The absorption of core-shell metallic NPs was not included in these calculations and is therefore considered parasitic absorption. Consequently, the electron-hole pair generation input for the electrical simulations is exclusively attributed to the perovskite materials within the absorber layers, ensuring accurate results for electrical calculations that depend on the electron-hole generation rate.

Following the calculation of the total electron-hole pair generation rates from Eqs. (7) and (8), the electrical characteristics were determined by solving the Poisson equation. Electrical calculations were initially performed for each sub-cell individually and then extended to the complete tandem structure, as illustrated in Fig. 2. These calculations were subsequently repeated with the inclusion of metallic core-shell NPs to facilitate a comparative analysis.

The Poisson equation is critical for understanding the electrical behavior of the AP-TSC under sunlight irradiation, specifically its impact on current-voltage and power-voltage curves. In this section, semiconductor physics was utilized to calculate the electrical properties of the tandem structure under the most accurate conditions using the FEM. Auger recombination and trap-assisted recombination mechanisms were incorporated to model the recombination process in the proposed structure. Electron-hole generation rates derived from Eqs. (7) and (8) were used as user-defined generation rates in the MAPb₀.₅Sn₀.₅I₃ and Cs₀.₂FA₀.₈Pb(I₀.₇Br₀.₃)₃ layers, with various doping levels evaluated in the targeted layers. The Poisson equation, represented by Eq. (9):

$$\nabla .( - {\varepsilon _0}\varepsilon \nabla V)=\rho$$,

9 $$\rho =q(p - n+N_{D}^{+} - N_{A}^{ - })$$

In Eq. (9), *V* denotes the electrostatic potential, *q* represents the elementary charge. The charge density *ρ* is determined, where *p* and *n* represent the concentrations of holes and electrons, respectively, while $\:{N}_{D}^{+}$ and $\:{N}_{A}^{-}$ denote the concentrations of ionized donors and acceptors. Solving the Poisson equation provides crucial insights into the electrical characteristics of each PSC sub-cell and the overall tandem structure, especially when under photon radiation. In addition to solving the Poisson equation, it is imperative to address the carrier continuity equations for electrons and holes. These equations are essential for understanding the transport and behavior of charge carriers within the AP-TSCs. Equations (10) and (11) represent the carrier continuity equations for electrons and holes, respectively,

10 $$\nabla .{J_n}+G - R=0$$

11 $$- \nabla .{J_p}+G - R=0$$

In Eq. (10), $\:{J}_{n}\:$represents the current density for electron, while ***G*** and ***R*** denote the electron-hole generation rate and recombination rate, respectively. Similarly, in Eq. (11), $\:{J}_{p}$ represents the current density for hole.

12 $${J_n}=qn{\mu _n}E+q{D_n}\nabla n$$

13 $${J_p}=qp{\mu _p}E - q{D_p}\nabla p$$.

In these equations, $\:{\mu\:}_{n}$ and $\:{\mu\:}_{p}$ denote the mobility of electron and hole carriers, respectively, $\:{D}_{n}$ and $\:{D}_{p}$ represent diffusion coefficients of electron and hole carriers. For calculating PCE (***η***) and fill-factor (FF) below equations will be used:

14 $$\eta =\frac{{{P_{\hbox{max} }}}}{{{P_{in}}}}=\frac{{FF \times {V_{OC}} \times {J_{SC}}}}{{{P_{AM1.5G}}}}$$

15 $$FF=\frac{{{P_{\hbox{max} }}}}{{{V_{OC}} \times {J_{SC}}}}$$

In these equations, $\:{P}_{max}$ is the maximum input power, and finally, $\:{V}_{oc}\:\text{a}\text{n}\text{d}\:{J}_{sc}$ are the open-circuit voltage and short-circuit current density, respectively. Finally, after current matching employment, the V_oc_ of tandem solar cell will be equal to Eqs. (16),

16 $${V_{oct}}={V_{ocn}}+{V_{ocw}}$$

In Eq. (16), V_oct_ is open-circuit voltage of tandem structure and V_ocn_ is open circuit voltage of NBG bottom sub cell and V_ocw_ is open-circuit voltage of WBG top sub cell. This equation is valid when the series connection is applied between sub cells.

**Fig. 3:**
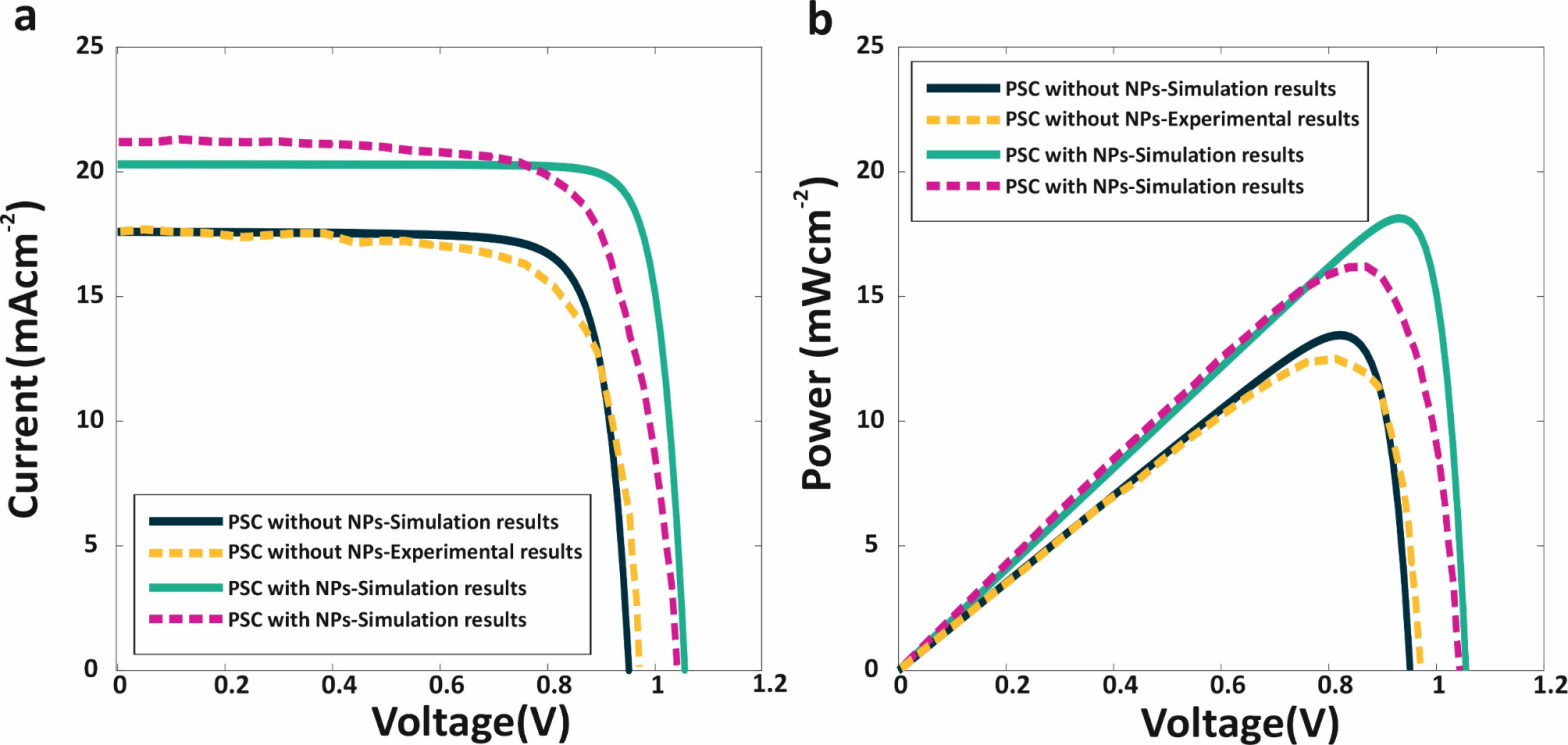
The validation of simulation method for bare PSC and NP embedded PSC. (a) *J* − *V* and (b) *P* − *V* curves for PSC mentioned in^66^. The bold dark-blue line obtained based on the simulation method, and the dashed yellow line is the experimental results for PSC. The bold green line obtained based on the simulation method, and the dashed magenta line is the experimental results for PSC with NPs embedded in absorber layer.

### B. Validation of simulation results and accuracy

Constructing a simulated model for the structure proves advantageous in designing efficient AP-TSCs, especially considering the limitations posed by laboratory resources and the time-intensive nature of fabrication. To enhance the precision of calculations and achieve more robust results, this research specifically employed the 3D-FEM for both optical and electrical simulations. This comprehensive approach ensures a more accurate representation of the complexities involved in AP-TSCs.

To validate the accuracy of our simulation model, the results obtained from 3D-FEM were compared with experimental data reported in^66^. The comparison was conducted using key metrics such as *J-V* and *P-V* curves of PSCs with and without embedded NPs, as illustrated in Fig. 3, and summarized in Table 2. This comparison demonstrates excellent agreement with the experimental data, confirming the accuracy and reliability of our model. Such validation underscores the robustness of our simulation framework and its capability to predict the optical and electrical behavior of PSCs and AP-TSCs reliably.

For instance, without Au@TiO₂ core-shell NPs (where Au is the core and TiO_2_ is the shell), the simulated values were *J*_*sc*_ = 17.66 mAcm^−2^, *V*_*oc*_ = 1.01 V, and *η* = 13.71%, while with Au@TiO₂ NPs, the values improved to *J*_*sc*_ = 20.3 mAcm^− 2^, *V*_*oc*_ = 1.05 V, and *η* = 16.87%. In comparison, the experimental results were *J*_*sc*_ = 17.40 mAcm^−2^, *V*_*oc*_ = 0.98 V, and *η* = 12.59% without NPs, and *J*_*sc*_ = 21.68 mAcm^−2^, *V*_*oc*_ = 1.04 V, and *η* = 16.88% with Au@TiO₂ NPs. The efficiency mismatch between simulation and experiment was only 1.12% without NPs and 0.01% with the presence of Au@TiO_2_ NPs. This high level of agreement, particularly with the inclusion of radiative effects from metallic NPs, underscores the robustness reliability of our 3D-FEM investigation.

**Table 2:** Comparison with simulation result and experimental results based on^66^.

Structure	J_sc_(mA/cm^2^)	V_oc_(V)	Efficiency (%)	Efficiency mismatch (%)
**Without NPs-Simulation**	17.66	1.01	13.71	1.12
**Without NPs-Experimental**	17.40	0.98	12.59	-
**With Au@TiO**_**2**_ **NPs-Simulation**	20.30	1.05	16.87	0.01
**With Au@TiO**_**2**_ **NPs-Experimental**	21.68	1.04	16.88	-

**Table 3:** Electrical parameters used for investigation of proposed all-perovskite tandem structure.

Parameter	SnO_2_	Cs_0.2_FA_0.8_Pb(I_0.7_Br_0.3_)_3_	MoO_3_	ITO	C_60_	MAPb_0.5_Sn_0.5_I_3_	WS_2_	PEDOT: PSS
**Bandgap (eV)**	3.6	1.75	3	3.5	1.7	1.17	1.8	1.6
**Electron affinity(eV)**	3.3	3.9	2.5	4	4	4.21	3.95	3.95
**dielectric permittivity**	9	10	2.5	10	4.2	13.6	13.6	3
**CB effective density of states (cm** ^**− 3**^ **)**	2.2 × 10^18^	1 × 10^19^	2.2 × 10^18^	2.2 × 10^18^	2.2 × 10^18^	1 × 10^19^	1 × 10^18^	2.2 × 10^18^
**VB effective density of states (cm** ^**− 3**^ **)**	1.8 × 10^19^	1.8 × 10^19^	1.8 × 10^19^	1.8 × 10^19^	1.8 × 10^19^	1.8 × 10^19^	2.4 × 10^19^	1.8 × 10^19^
**Electron mobility** **(cm**^**2**^. **V**^**− 1**^. **s**^**− 1**^**)**	100	50	25	20	0.08	100	100	4.5 × 10^− 2^
**Hole mobility** **(cm**^**2**^. **V**^**− 1**^. **s**^**− 1**^**)**	25	50	100	10	3.5 × 10^− 3^	25	100	4.5 × 10^− 2^
**Doping concentration of donors**,** ND (cm**^**− 3**^**)**	1 × 10^17^	1 × 10^9^	-	1 × 10^21^	1 × 10^21^	-	-	-
**Doping concentration of acceptors**,** NA (cm**^**− 3**^**)**	-	1 × 10^9^	9 × 10^15^	-	-	1 × 10^9^	-	9 × 10^15^

## Results and discussion

In this section, the results obtained from the comprehensive analysis of the AP-TSC are presented and discussed. The findings are organized into several key areas to provide an in-depth understanding of the optical and electrical characteristics of both the WBG and NBG sub-cells, as well as the overall performance of the tandem structure. Initially, the optical and electrical investigations of the WBG sub-cell are examined, followed by those of the NBG sub-cell. Subsequently, the performance of the proposed carbon-based AP-TSC is analyzed, with a focus on the benefits and limitations associated with carbon-based materials. The discussion then progresses to the alignment of the band diagram of the top and bottom sub-cells, which is critical for optimizing tandem performance. The impact of temperature on device efficiency is also explored, along with a sensitivity analysis of input parameters to assess their effects on performance. Finally, the challenges encountered when transitioning from simulation to real-world applications are addressed. This structured presentation aims to provide a thorough evaluation of the AP-TSC’s performance and the factors influencing its efficiency.

### A. Optical and electrical investigation of WBG sub-cell

**Fig. 4:**
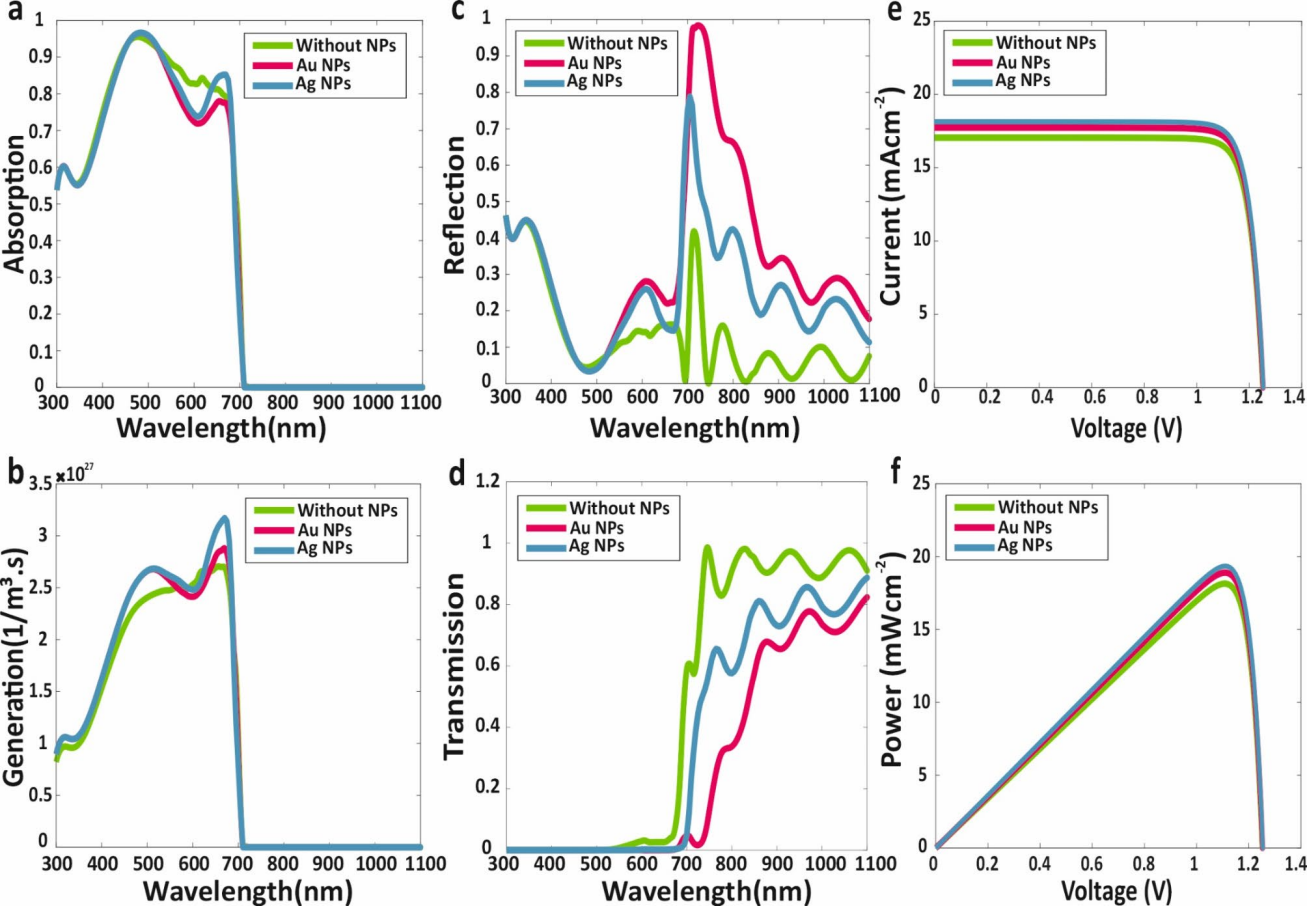
(**a**) Absorption (**b**) Electron-hole generation (**c**) Reflection (**d**) Transmission Spectra for proposed WBG sub-cell with absence and presence of Ag and Au metallic core-shell NPs. (**e**) Current-Voltage curve, and (**f**) Power-Voltage curve for WBG sub-cell without and with of metallic core-shell NPs.

The investigation commences with an analysis of each sub-cell individually as SJ-PSCs. Initially, the focus is placed on the WBG sub-cell of the proposed tandem structure, as depicted in Fig. 1 (top-cell section). The specific configuration examined includes layer thicknesses of 65 nm for the ITO AR layer, 100 nm for the SnO_2_ ETL, 1000 nm for the Cs_0.2_FA_0.8_Pb(I_0.7_Br_0.3_)_3_ perovskite absorber layer, 12 nm for the WS_2_ TMD layer, 150 nm for the MoO_3_ HTL, 17 nm for the ITO back-contact layer, 70 nm for the height and width of metallic NPs, 500 nm for the thickness of metallic NPs, 5 nm for the dielectric nano-shell of metallic NPs and 20 nm distance of NPs from the TMD top surface. Maxwell’s equations were solved to obtain the electric field distribution within this structure. Subsequently, the optical characteristics of proposed SJ-WBG sub-cell structure, including absorption, electron-hole generation, transmission and reflection were determined. The process was then repeated with the incorporation of metallic core-shell NPs in the absorber layer for comparative analysis. Figure 4 presents the spectra for optical characteristics of the WBG sub-cell without and with Ag and Au metallic core-shell NPs: (a) absorption, (b) electron-hole generation, (c) reflection, (d) transmission; as well as (e) the current-voltage and f) the power-voltage curves for the WBG sub-cell in both scenarios.

As shown in Fig. 4a and b, the incorporation of metallic core-shell NPs significantly enhances the electron-hole generation within the proposed structure, thereby improving the short-circuit current density and overall efficiency of the WBG. Among the metallic core-shell NPs, Ag NPs demonstrate superior performance compared to Au NPs. Furthermore, it is observed that absorption and electron-hole generation in the WBG-sub-cell cease beyond 700 nm, with no additional electron-hole pairs generated at longer wavelengths. As shown in Fig. 4c and d, photon absorption beyond 700 nm is attributed to the NBG sub-cell.

Following the optical analysis, electrical calculations were performed using the parameters detailed in Table 3. The short-circuit current density *J*_*sc*_, open-circuit voltage *V*_*oc*_, efficiency *η* and fill factor FF for WBG sub-cell were determined to be 17.05 mAcm^− 2^, 1.25 V, 18.14% and 85.14%, respectively. With the addition of Au core-shell NPs, these parameters improved to 17.73 mAcm^− 2^, 1.25 V, 18.90%, and 85.27%. The integration of Ag core-shell NPs further enhanced the performance, yielding values of 18.14 mAcm^− 2^, 1.25 V, 19.35%, and 85.33%. The *J-V* curve for the WBG sub-cell during individual investigation is depicted in Fig. 4e, while Fig. 4f provides a comparison of the P-V spectrum for the same sub-cell. A comprehensive summary of all electrical characteristics for the WBG sub-cell is presented in Table 4.

**Table 4:** Electrical characteristics of WBG and NBG sub-cells investigated individually without and with presence of metallic core-shell NPs.

	Parameter	J_sc_ (mAcm^− 2^)	V_oc_ (V)	η (%)	FF (%)
**WBG**	**Without core-shell NPs**	17.05	1.25	18.14	85.14
	**With Au core-shell NPs**	17.73	1.25	18.90	85.27
	**With Ag core-shell NPs**	18.14	1.25	19.35	85.33
**NBG**	**Without core-shell NPs**	27.51	0.73	16.10	80.01
	**With Au core-shell NPs**	35.51	0.74	20.89	79.48
	**With Ag core-shell NPs**	35.95	0.74	21.15	79.49

**Fig. 5:**
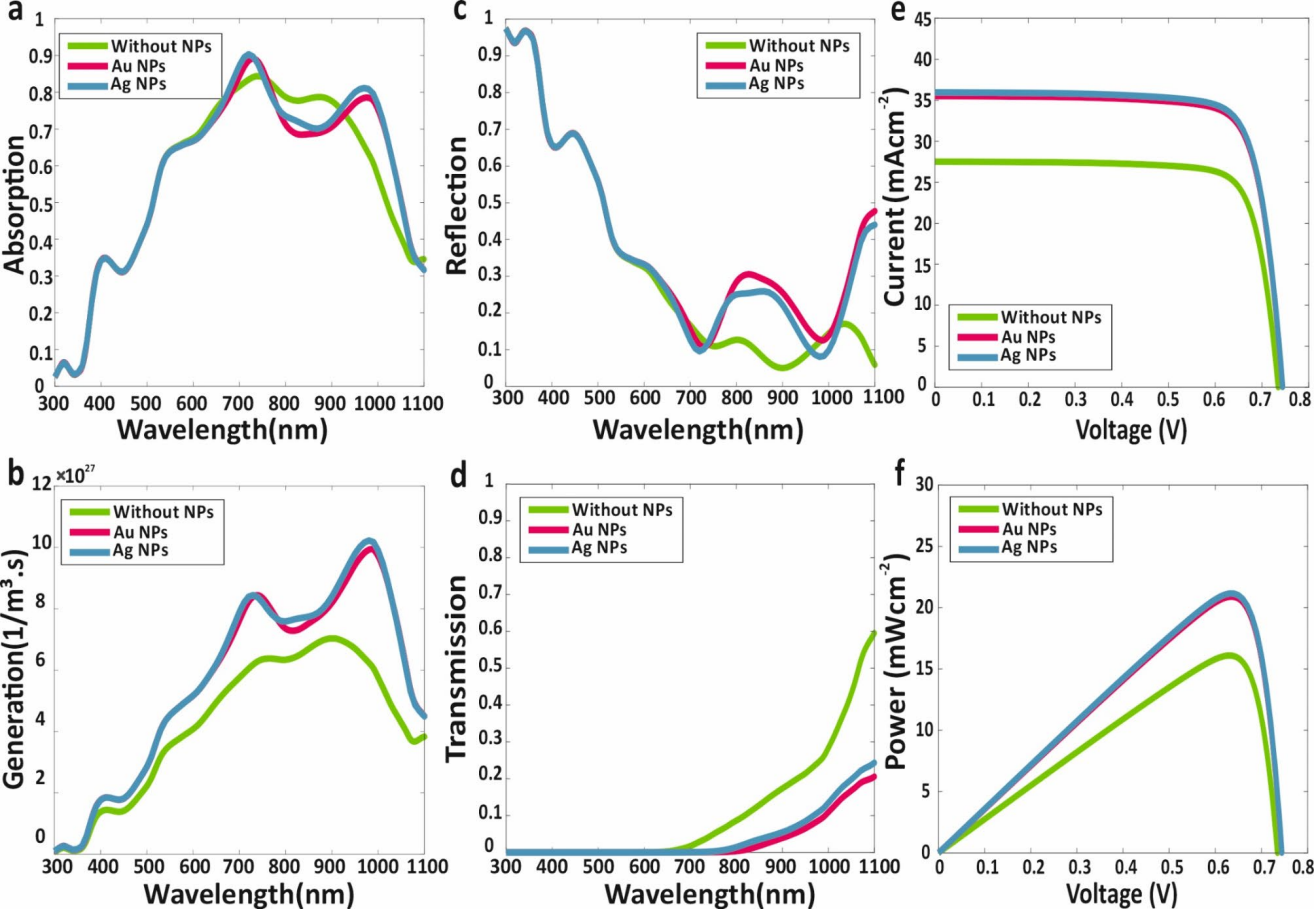
(**a**) Absorption (**b**) Electron-hole generation (**c**) Reflection (**d**) Transmission spectrums for proposed NBG sub-cell for absence and presence of Ag and Au metallic core-shell NPs. (**e**) Current-voltage curve, and (**f**) Power-voltage curve for NBG sub-cell without and with of metallic core-shell NPs.

### B. Optical and electrical investigation of NBG sub-cell

Following the investigation of the WBG sub-cell, the NBG sub-cell was examined in detail, with both optical and electrical characteristics evaluated in the presence and absence of metallic core-shell NPs. The layer structure for the SJ-NBG sub-cell is depicted in Fig. 1 (NBG sub-cell), with layer thicknesses as follows: 17 nm for the ITO AR layer, 100 nm for the C_60_ ETL, 500 nm for the $\:{\text{M}\text{A}\text{P}\text{b}}_{0.5}{\text{S}\text{n}}_{0.5}{\text{I}}_{3}$ perovskite absorber layer, 50 nm for the PEDOT: PSS HTL, 80 nm for the carbon back-contact layer, 120 nm for the height and width of metallic NPs, 180 nm for the thickness of metallic NPs, 5 nm for the dielectric nano-shell of metallic NPs, and 20 nm for the distance of metallic core-shell NPs and top surface of HTL. The principal optical characteristics of the NBG sub-cell are presented in Fig. 5.

As illustrated in Fig. 5a, absorption spectrum extends up to1100 nm, with enhanced absorption at higher wavelengths observed upon embedding metallic NPs in the MAPb₀.₅Sn₀.₅I₃ layer. Consequently, as shown in Fig. 5b, electron-hole pair generation occurs at higher wavelengths, with Ag core-shell NPs demonstrating superior performance compared to Au types. The increased electron-hole pair generation resulting from the presence of embedded core-shell metallic NPs leads to an improved photocurrent and, consequently, an enhanced efficiency of the NBG sub-cell.

Following the optical analysis, electrical calculations were performed. The short-circuit current density *J*_*sc*_, open-circuit voltage V_oc_, efficiency *η*, and fill factor FF for the NBG sub-cell were found to be 27.51 mAcm^− 2^, 0.73 V, 16.10% and 80.01%, respectively. Upon incorporating Au core-shell NPs, these parameters improved to 35.51 mAcm^− 2^, 0.74 V, 20.887% and 79.48%. Further enhancement was observed with the addition of Ag metallic NPs, resulting in values of 35.95 mAcm^− 2^, 0.74 V, 21.15% and 79.49%. The *J-V* curve for the NBG sub-cell during individual investigation is depicted in Fig. 5e, while Fig. 5f shows the *P-V* curve for the same sub-cell. A summary of all electrical parameters is provided in Table 4.

It was observed that incorporating metallic core-shell NPs into the absorber layer of sub-cells enhances the photocurrent and efficiency. However, when the WBG sub-cell is stacked on the NBG sub-cell, the absorption characteristics of bottom sub-cell are influenced by the shape and size of metallic core-shell NPs in the top sub-cell. Wider metallic core-shell NPs in the WBG absorber layer reduce photon transmission to the NBG sub-cell, highlighting the importance of NP size and shape. Additionally, local field enhancement is more effective at sharp edges of the NPs compared to other locations.

**Fig. 6:**
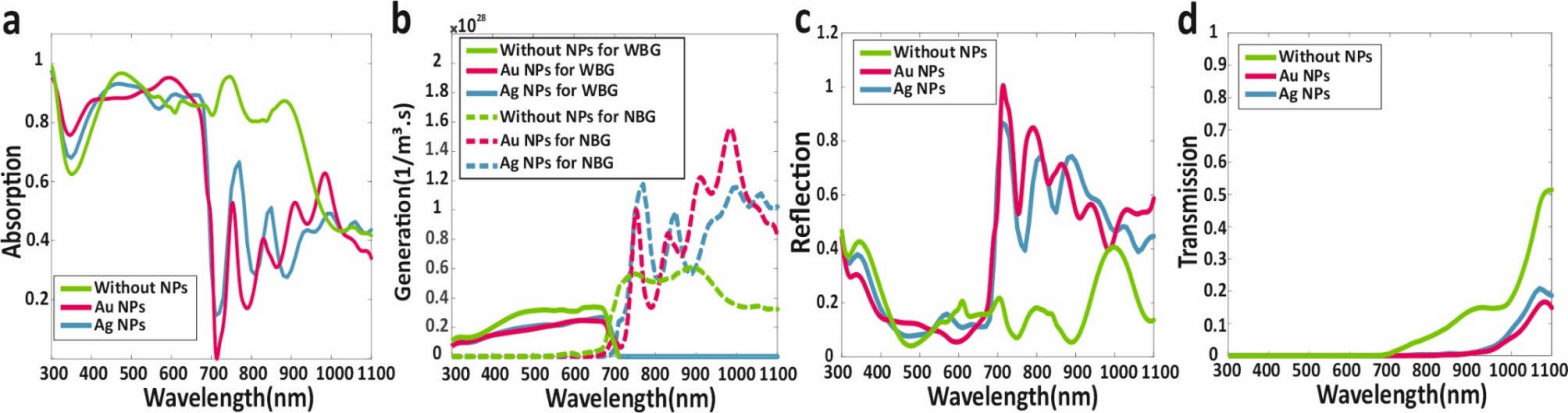
(**a**) Absorption, (**b**) Electron-hole generation rate, (**c**) Reflection, and (**d**) Transmission of proposed AP-TSC.

**Fig. 7:**
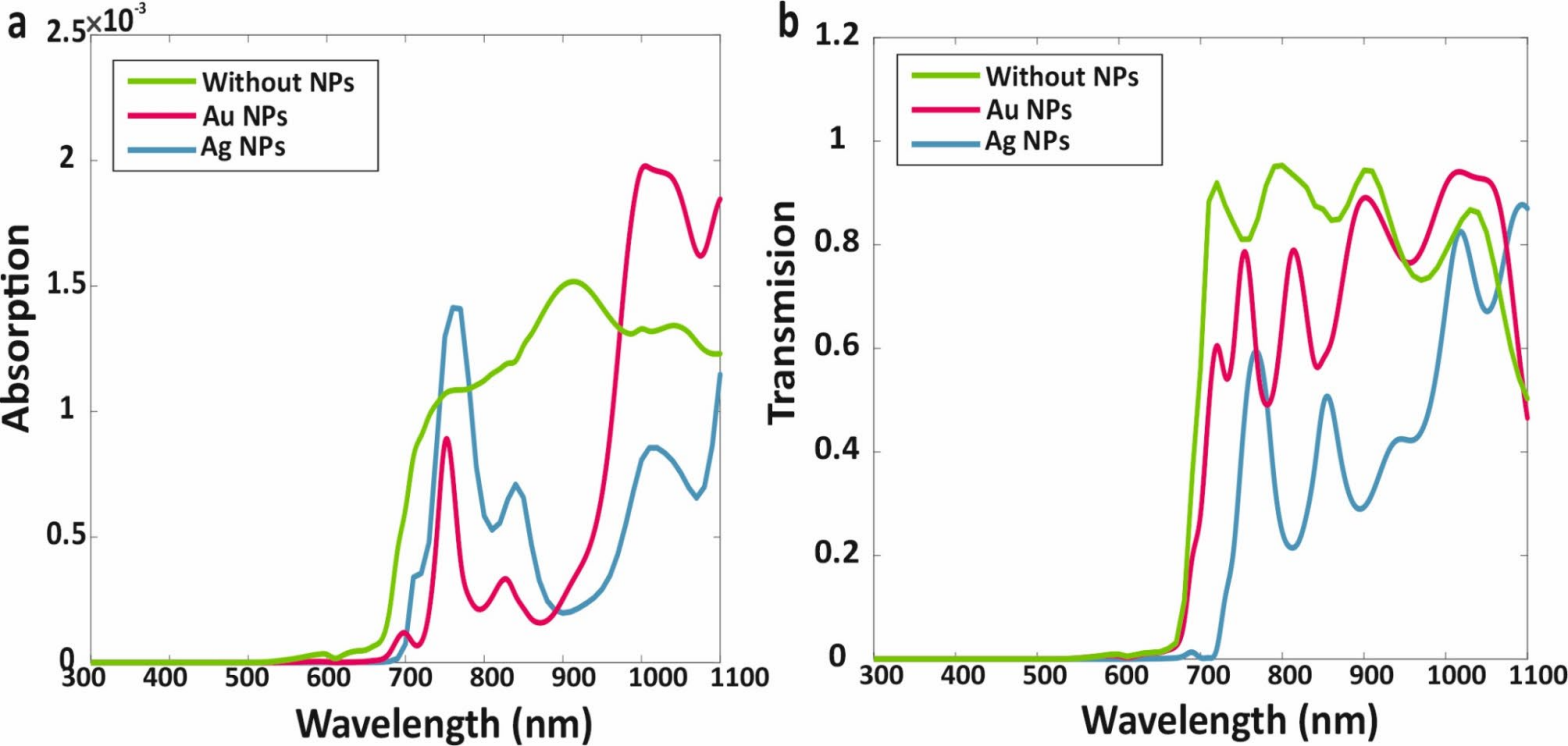
(**a**) Absorption and (**b**) Transmission of ITO ICL in the proposed AP-TSC.

### C. Optical and electrical investigation of proposed carbon-based AP-TSC

After the individual analysis of each sub-cell, the WBG sub-cell was configured as the top sub-cell, with the NBG sub-cell positioned as the bottom sub-cell, and an ITO ICL was introduced to form the AP-TSC. The layer thicknesses are summarized in Table 1. To assess the optical characteristics of the tandem structure, using the 3D-FEM method, and the results are presented in Fig. 6. Figure 6 illustrates the main optical characteristics of the proposed AP-TSC with and without core-shell metallic NPs for optimal conditions. Notably, the AP-TSC structure exhibits a broad absorption wavelength range of 300–100 nm, which contributes to enhanced electron-hole pair generation and improved efficiency, as depicted in Fig. 6a and b. Figure 6c and d show the reflection and transmission spectra for the tandem structure.

Given the importance of the ICL, its absorption and transmission characteristics were also investigated. As shown in Fig. 7a, the absorption of ITO was found to be weak, allowing a significant number of remaining photons from the WBG sub-cell’s absorption to pass through the ITO layer and reach the NBG sub-cell. The transmission spectrum of the ICL was analyzed and illustrated in Fig. 7b, revealing that a substantial portion of photons passed through the ICL with minimal loss. This high transmission efficiency ensures that sufficient light reaches the NBG sub-cell, enhancing overall device performance.

**Fig. 8:**
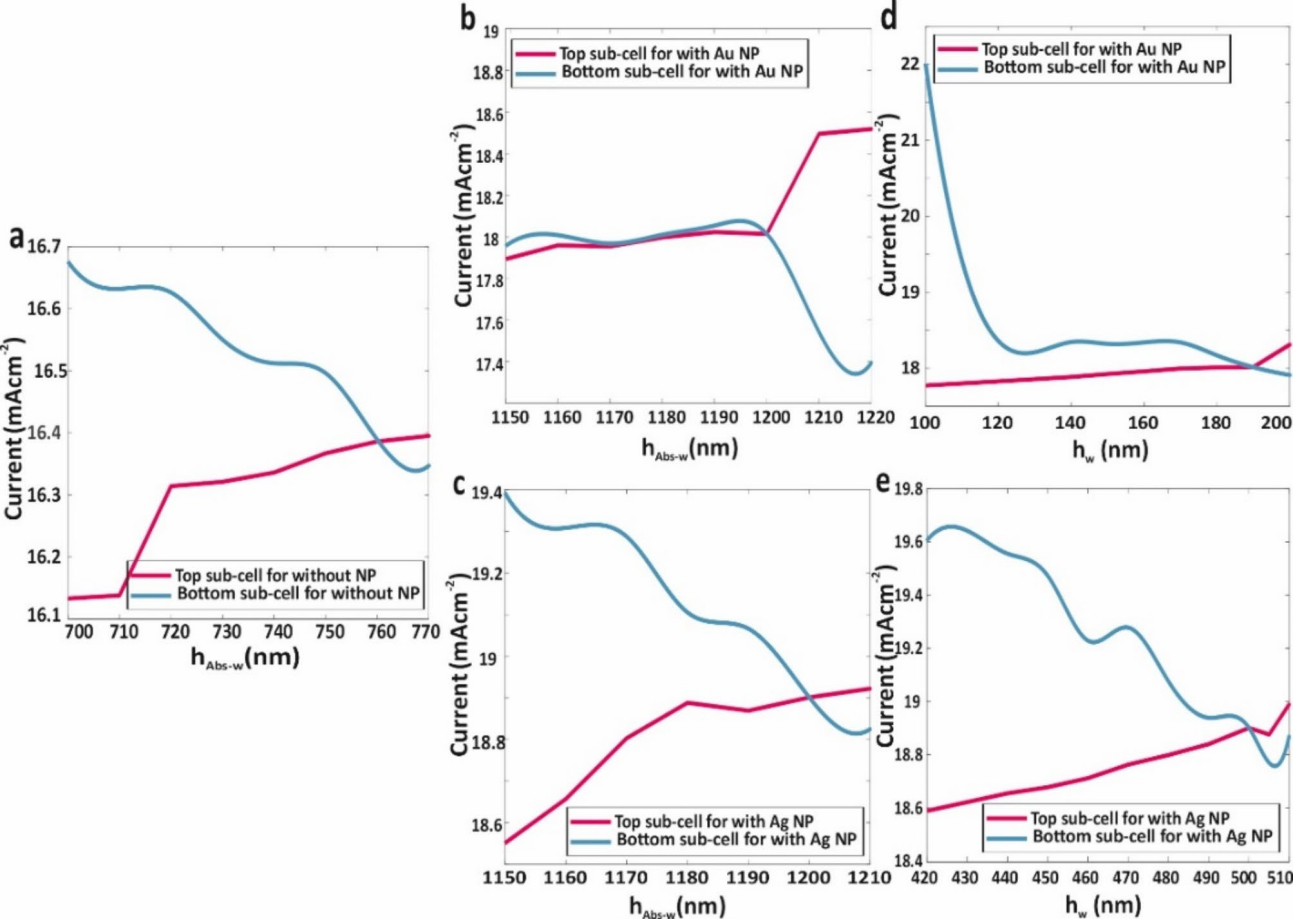
(**a**) Current matching between sub-cells of proposed AP-TSC without any NPs. Current matching between sub-cells of proposed AP-TSC with variation of (**b**) the WBG absorber layer thickness in presence of Au core-shell NPs, (**c**) the WBG absorber layer thickness in presence of Ag core-shell NPs, **(d)** the thickness of Au core-shell NPs embedded in WBG absorber layer, and (**e**) the thickness of Ag core-shell NPs embedded in WBG absorber layer. (h_Abs−n_ = 600 nm)

Figure 8 depicts the current matching mechanism without and with the incorporation of metallic core-shell NPs. In Fig. 8a, the current matching mechanism for the WBG and NBG sub-cells is presented without any metallic NPs. Upon embedding metallic core-shell NPs, as shown in Fig. 8b and e, the local field enhancement within the absorber layers significantly improved the optical and electrical properties of the proposed AP-TSC necessitating a recalibration of the previous current matching mechanism. The short-circuit current density of the sub-cell is now influenced by several parameters, including the thickness and size of the metallic core-shell NPs. Figure 8b illustrates the current matching with varying WBG absorber thickness in the presence of Au core-shell NPs, while Fig. 8c a similar variation with Ag core-shell NPs. Moreover, Fig. 8d and e demonstrate how the thickness of the Au and Ag core-shell NPs embedded in the WBG absorber are varied to ensure accurate current matching. The thickness of plasmonic NPs in the WBG absorber is particularly critical, as the short-circuit current density of the tandem solar cell is closely tied to the WBG absorber layer’s performance.

Following the current-matching mechanism and calculation of the Ap-TSC’s electrical properties, the following values were obtained for the structure without metallic NPs: short-circuit current density (*J*_*sc*_) of 16.39 mAcm^− 2^, open-circuit voltage (*V*_*oc*_) of 1.97 V, efficiency (*η*) of 27%, fill factor (FF) of 83.4%, NBG absorber thickness (*h*_*Abs−n*_) of 600 nm, WBG absorber thickness (*h*_*Abs−w*_) of 760 nm, and metallic core-shell NP thickness (*h*_*w*_) of zero.

**Fig. 9:**
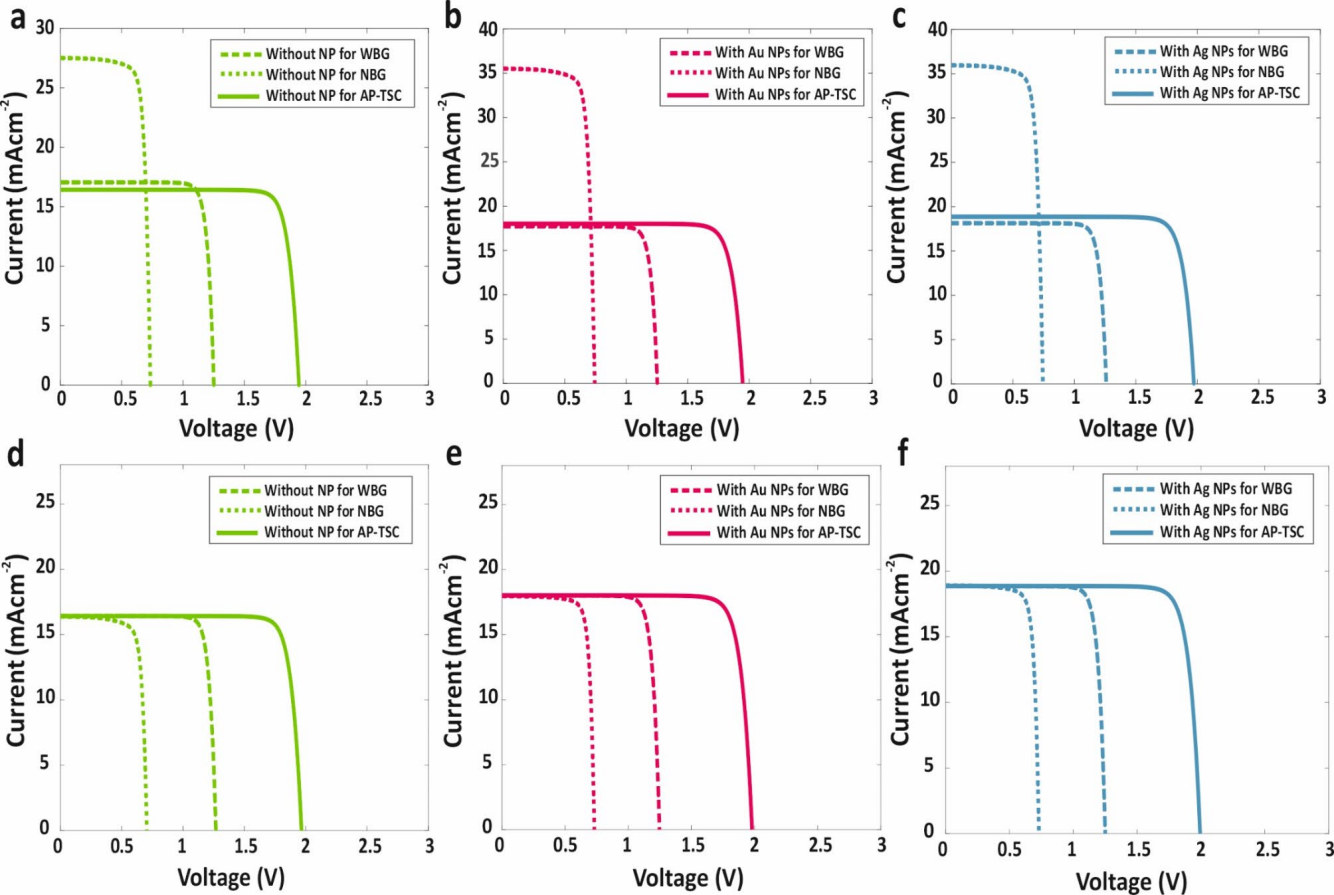
*J-V* curves for standalone WBG and NBG sub-cells and AP-TSC for: (**a**) without NPs. (**b**) with presence and absence of Au core-shell metallic NPs. (**c**) With presence and absence of Ag core-shell metallic NPs. J-V curve for wide and narrow band-gap sub-cells and AP-TSC after current matching for: (**d**) Without NPs. (**e**) With presence and absence of Au core-shell metallic NPs. (**f**) With presence and absence of Ag core-shell metallic NPs.

**Table 5:** Electrical properties of proposed AP-TSC.

		J_sc_ (mA/cm^2^)	V_oc_ (V)	η (%)	FF (%)	Current mismatch (mA/cm^2^)
**Without NPs**	**Wide band-gap sub-cell**	16.39	1.27	17.8	85.51	-
	**Narrow band-gap sub-cell**	16.386	0.705	9.1	78.77	-
	**Tandem Structure** **(Based on carbon back contact)**	16.39	1.97	26.9	83.4	0.004
	**Tandem Structure** **(Based on Au back contact)**	16.54	2.05	29.17	85.9	
**Au NPs**	**Wide band-gap sub-cell**	18.01	1.25	18.97	84.25	-
	**Narrow band-gap sub-cell**	18.01	0.73	10.7	81.36	-
	**Tandem Structure**	18.01	1.98	29.67	83.2	0
	**Tandem Structure without nano-shell**	17.8	1.98	29.3	83.13	0.007
**Ag NPs**	**Wide band-gap sub-cell**	18.90	1.25	19.99	84.6	-
	**Narrow band-gap sub-cell**	18.90	0.73	11.12	80.24	-
	**Tandem Structure**	18.90	1.98	31.12	83.15	0.003
	**Tandem Structure without nano-shell**	18.2	1.98	29.94	83.08	0.006

Incorporating Au metallic core-shell NPs improved these values to 18.01 mAcm^− 2^, 1.98 V, 29.67%, 83.2%, 430 nm, 1200 nm, and 190 nm. For Ag metallic core-shell NPs, the results were further enhanced, yielding 18.90 mAcm^− 2^, 1.98 V, 31.12%, 83.15%, 450 nm, 1200 nm, and 500 nm. When dielectric nano-shells around the metallic NPs were excluded, the corresponding values for Au (Ag) were slightly reduced to 17.8 (18.2) mAcm^− 2^, 1.98 V, 29.3 (29.94)%, 83.13 (83.8)%, 430 (450) nm, 1200 nm, and 195 (470) nm, respectively. Figure 9a and b, and 9c illustrate the *J-V* curves for the SJ-WBG, SJ-NBG, and the AP-TSC, respectively, with each curve comparing the structure without metallic NPs, with Au core-shell NPs, and with Ag core-shell NPs. Additionally, Fig. 9d and e, and 9f present the *J-V* curves after current matching for these configurations.

The choice of back contact material plays a crucial role in optimizing the device performance, particularly by influencing the energy barrier for charge extraction. Our study examined the effect of different work functions for Au and carbon back contacts. The Au contact, with a work function of 5.1 eV, resulted in an efficiency of 29.17% without metallic NPs. In comparison, the carbon contact, with a slightly lower work function of 5 eV, offered a marginally reduced efficiency but demonstrated superior overall stability. A detailed comparison of the electrical characteristics for these configurations is provided in Table 5.

### D. Band alignment of top and bottom sub-cells

One critical factor in the performance of TSCs is the alignment of the energy bands between the WBG top sub-cell and the NBG bottom sub-cell. Proper band alignment ensures efficient charge transfer across the ICL, minimizing recombination and energy losses. In our carbon-based AP-TSC, the ETLs and HTLs have been carefully selected to achieve optimal band alignment.

In the context of stacking layers within an AP-TSC, the recombination layer is a crucial component. This layer electrically connects both sub-cells in series. When an electron-hole pair is generated within a sub-cell, one carrier type is extracted through the external contact of that sub-cell (negative terminal). To maintain overall charge neutrality and facilitate efficient charge extraction, the recombination layer must provide an effective recombination pathway for the remaining hole, which recombines with a photogenerated electron from the other sub-cell. The resulting hole is then extracted at the external contact of the second sub-cell (positive terminal). This process allows for the transport of one electron through the entire tandem stack, albeit at a higher voltage due to the series connection.

**Fig. 10:**
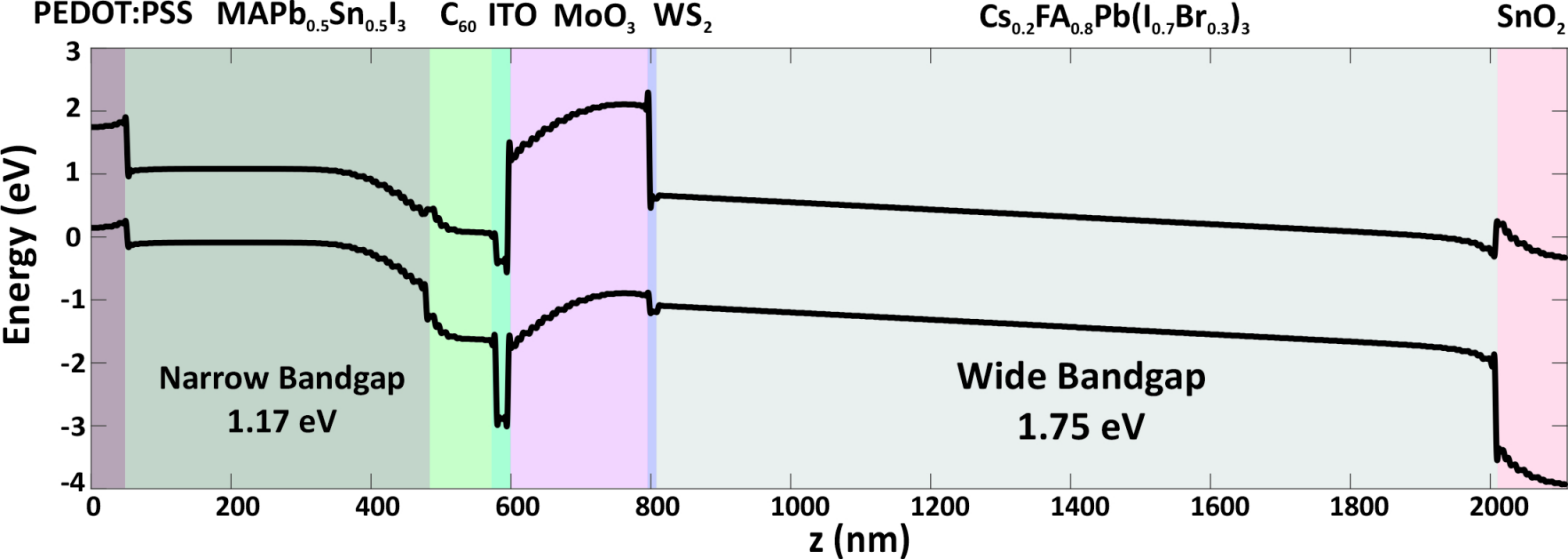
Band diagram of proposed carbon-based AP-TSC.

Figure 10 illustrates the conduction band of ITO and the valence band of MoO₃ at the ITO recombination layer coming close to each other. This proximity results in the formation of a tunnel junction, which facilitates the recombination of electrons from the NBG sub-cell with holes from the WBG sub-cell. This electron-hole recombination establishes a series connection, contributing to the design of the tandem structure. The recombination layer must exhibit excellent selectivity for the respective charge carriers, favorable energetic alignment to enable lossless photovoltage addition, and minimal series resistance. Additionally, it must maintain high optical transparency, particularly in the near-infrared range. Figure 7b shows that beyond the absorption range of the top sub-cell, transmission through the ITO layer increases, allowing the remaining photons to reach the NBG sub-cell.

To effectively capture a broader range of the light spectrum, it is essential to ensure precise optical alignment of the absorber layers. Well-aligned bandgaps mitigate the efficiency limitations inherent in tandem structures. The detailed balance limit for a two-terminal perovskite tandem structure with absorber bandgaps of 1.17 eV and 1.75 eV is 36%, highlighting the significant potential of such structures to achieve high efficiencies.

**Fig. 11:**
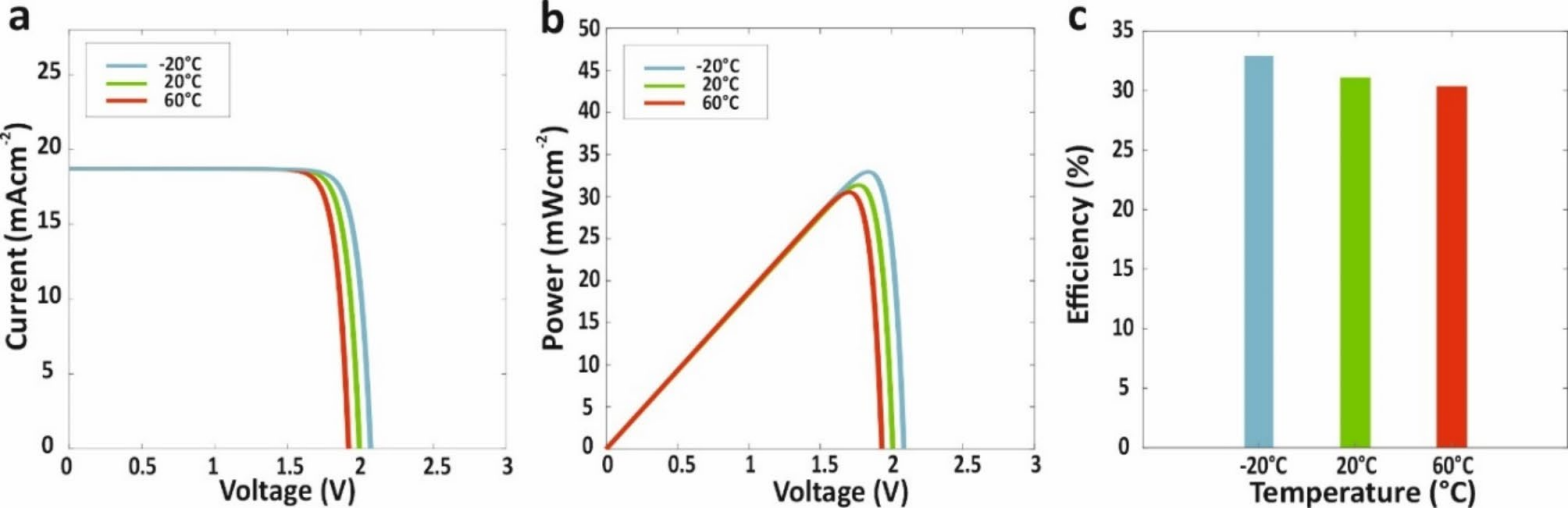
Effect of variation of temperature on (**a**) J-V curve, (**b**) P-V curve, and efficiency of proposed carbon-based AP-TSC with presence of Ag NPs.

### E. Impact of temperature on device performance

To evaluate the real-world performance of the carbon-based AP-TSCs, temperature-dependent simulations were conducted. These simulations modeled device performance across a range of temperatures, accounting for variations in charge carrier mobility, band gaps, recombination rates, and other temperature-sensitive parameters. The aim was to gain insight into how ambient temperature affects the efficiency and stability of the AP-TSCs under different environmental conditions.

The temperature-dependent simulations reveal a clear impact of ambient temperature on the performance of the carbon-based AP-TSCs. As shown in Fig. 11, the *J-V* and *P-V* curves at various temperatures demonstrate that increasing temperature leads to a noticeable decline in key performance metrics. Specifically, higher temperatures result in a reduction in open-circuit voltage and the overall efficiency. This decline is attributed to increased carrier recombination, reduced charge carrier mobility, and band gap narrowing at higher temperatures—effects that are well-documented in PSCs. Conversely, at lower temperatures, open-circuit voltage and efficiency improve due to the reduction in thermal recombination losses, leading to enhanced device performance^67^.

The variation of these parameters with temperature confirms the sensitivity of AP-TSCs to environmental conditions, which must be considered for real-world applications. The detailed analysis of the *J-V* and *P-V* curves across the temperature range provides a comprehensive understanding of how thermal effects influence the electrical behavior of the AP-TSCs. These findings underscore the importance of thermal management in improving the stability and performance of AP-TSCs under varying operating conditions.

While temperature effects are a significant factor in AP-TSC performance, ensuring the robustness of the model against design variations is equally crucial. In the next section, we present a sensitivity analysis to assess how small changes in design parameters, such as NP thickness, influence device performance.

### F. Sensitivity analysis of input parameters

Sensitivity analysis is a crucial step in validating simulation models, as it allows us to assess how variations in input parameters influence overall device performance, ensuring the model’s reliability under different fabrication conditions. In this study, we performed a sensitivity analysis to evaluate the impact of small changes in metallic NP thickness on the key performance metrics of our carbon-based AP-TSCs.

To assess the sensitivity of the model, we varied the thickness of the metallic NPs in both the NBG and WBG sub-cells by ± 5% from the baseline values used in the simulations. We then observed how this variation affected the short-circuit current (Jsc) and overall PCE of the AP-TSCs.

The sensitivity analysis revealed that increasing or decreasing the NP thickness by 5% resulted in an approximate change of 0.1 mA/cm² in J_sc_, corresponding to only 0.52% of the total J_sc_. Similarly, the PCE showed negligible variation under these changes. These results suggest that the model is robust, as the device performance is minimally affected by small variations in NP thickness. This parameter was chosen for analysis due to its significant influence on the optical and electrical properties of AP-TSCs.

This minimal change in performance under varying NP thicknesses demonstrates that the AP-TSC design is tolerant of small deviations in fabrication processes, such as layer thickness variations or imperfections in NP integration. The robustness of the model suggests that the simulated design can be reliably translated into real-world manufacturing, where such variations are often unavoidable.

In summary, the sensitivity analysis indicates that small variations in the thickness of metallic NPs have a minimal effect on the key performance metrics of the AP-TSCs. This finding underscores the robustness of our simulation model and suggests that it can accommodate potential fabrication variations without significant loss of performance. This robustness is essential for ensuring the practical viability of the design in large-scale production.

While the sensitivity analysis demonstrates that the model is robust against small variations in design parameters, scaling up the AP-TSCs for real-world applications presents additional challenges. Beyond the precision of design, factors such as material uniformity, fabrication methods, and environmental stability must be considered to ensure the successful large-scale deployment of these devices.

### G. Challenges in scaling from simulation to real-world applications

While the simulations presented in this study offer promising insights into the performance potential of AP-TSCs, transitioning from simulation to large-scale, real-world applications introduces several challenges. These challenges stem from the need to maintain material quality, stability, and cost-effectiveness while ensuring the scalability of fabrication methods. In this section, we discuss the primary factors that must be considered to successfully scale up the proposed AP-TSC designs for industrial applications.

In simulations, material properties such as the uniformity of perovskite layers and precise NP distribution were assumed to be ideal. However, achieving such uniformity in large-scale production presents significant challenges. Defects, grain boundaries, and the non-uniform distribution of NPs can significantly affect device performance. Ensuring material consistency across large areas is critical for maintaining efficiency in real-world devices^68,69^.

Laboratory-scale techniques, such as spin-coating, are effective for small-scale solar cell production but are difficult to scale for larger areas. Industrial-scale fabrication methods, such as roll-to-roll processing, must be adopted for real-world applications. This transition can introduce variations in layer thickness and NP integration, potentially affecting device performance. Developing scalable and reproducible manufacturing processes is crucial for translating the results of our simulations into practical applications^70,71^.

Perovskite materials are known for their sensitivity to environmental factors such as moisture, UV light, and oxygen, which can lead to material degradation over time. Although our simulations model ideal conditions, real-world devices must contend with these stability issues. Effective encapsulation strategies and material optimizations are necessary to improve the long-term durability of AP-TSCs, particularly in outdoor environments. The use of carbon-based back contacts offers enhanced stability compared to metallic contacts, but further research is needed to address the long-term performance of these devices^72^.

Our temperature-dependent simulations indicate that higher ambient temperatures negatively affect the efficiency and open-circuit voltage of AP-TSCs. In real-world applications, large-area devices are exposed to a wider range of temperatures and humidity conditions, which can exacerbate thermal stress and performance degradation. Managing these environmental effects is crucial for maintaining device efficiency in varying climates^67^.

Cost considerations are a key factor when scaling up solar cell production. While silver NPs offer a more affordable alternative to gold, their large-scale use still presents cost challenges. Identifying cost-effective materials and optimizing the use of expensive resources will be essential for ensuring the commercial viability of AP-TSCs. Striking a balance between material costs and maintaining high efficiency is critical for large-scale deployment.

In summary, while our simulations offer valuable predictions for the potential performance of AP-TSCs, it is important to recognize the limitations inherent in simulation-based studies. Many real-world factors, such as temperature-induced reductions in mobility, bandgap narrowing, and long-term material degradation due to environmental exposure (e.g., moisture and UV light), are often not fully considered or accurately modeled. These factors can have a significant impact on the device’s long-term performance and efficiency. Therefore, while simulations provide an excellent starting point, experimental validation and optimization are essential to address these real-world challenges and ensure that AP-TSCs perform reliably under practical conditions.

**Conclusion.** In this research, optical and electrical investigation of an AP-TSC structure with 1.75 eV/1.17 eV bandgap structure with very thin ITO IC layer have been performed. Both of optical and electrical calculations has been done using 3D FEM method. in the proposed tandem structure, carbon back electrode instead of metal back electrode has been used to increase stability of the tandem structure and reduce fabrication hardness like thermal evaporation. To compensation of efficiency reductions and to reduce toxicity of pb based perovskite absorber layer, cubic plasmonic metallic NP have been embedded in absorber layers and electron-hole generation in both absorber layers has been enhanced. Also, to prevent direct contact between metallic NPs and perovskite materials, increase thermal and chemical stability and reduction of recombination on the surface of metallic NPs, 5 nm dielectric shell has been incorporated on the surface of metallic NPs. Before embarking on the core investigation of the tandem structure, we conducted a validation simulation to showcase the precision and dependability of our simulations. Noteworthy is the slight efficiency discrepancy observed during this validation simulation, particularly concerning the integration of metallic nanoparticles (NPs), which amounted to a mere 0.01%. first, optical and electrical investigations performed to each of sub-cells separately to observe effect of core-shell metallic NPs incorporation and then, complete AP-TSC tandem structure has been investigated. current matching mechanism performed with absence and presence of metallic cubic core-shell NPs and optimum performance for proposed tandem structure observed. finally, for both optical and electrical investigations, Ag NPs had better performance and by incorporation of cubic core-shell Ag NPs, 15.32% enhancement in short-circuit current of tandem structure (from 16.39 mA/cm^2^ to 18.901 mA/cm^2^) and 15.68% efficiency enhancement (from 26.9 to 31.12%) for overall efficiency of tandem structure observed. In a dedicated section, we examined the band alignment of the sub-cell. Furthermore, we conducted a thorough thermal investigation of the proposed tandem structure, demonstrating the robustness of the AP-TSC against climate changes. Finally, we extensively discussed sensitivity analyses related to input parameters and the challenges associated with large-scale fabrication of the proposed AP-TSC. this research opens door for usage of core-shell metallic NPs in AP-TSCs for efficiency and stability enhancement.

## Data Availability

The datasets generated during and/or analysed during the current study are available from the corresponding author on reasonable request.
